# COVID-19 classification using thermal images

**DOI:** 10.1117/1.JBO.27.5.056003

**Published:** 2022-05-18

**Authors:** Martha Rebeca Canales Fiscal, Victor Treviño, Luis Javier Ramírez Treviño, Rocio Ortiz López, Servando Cardona Huerta, Victor Javier Lara-Díaz, José Gerardo Tamez Peña

**Affiliations:** a Tecnologico de Monterrey, Escuela de Ingeniería y Ciencias, Monterrey, Nuevo León, México; b Tecnologico de Monterrey, Escuela de Medicina y Ciencias de la Salud, Monterrey, Nuevo León, México; c Tecnologico de Monterrey, The Institute for Obesity Research, Integrative Biology Unit, Monterrey, Nuevo Leon, México; d Tecnologico de Monterrey, Hospital Zambrano Hellion, San Pedro Garza García, Nuevo León, México

**Keywords:** coronavirus disease-19 classification, thermal videos, thermal images, machine learning

## Abstract

**Significance:**

There is a scarcity of published research on the potential role of thermal imaging in the remote detection of respiratory issues due to coronavirus disease-19 (COVID-19). This is a comprehensive study that explores the potential of this imaging technology resulting from its convenient aspects that make it highly accessible: it is contactless, noninvasive, and devoid of harmful radiation effects, and it does not require a complicated installation process.

**Aim:**

We aim to investigate the role of thermal imaging, specifically thermal video, for the identification of SARS-CoV-2-infected people using infrared technology and to explore the role of breathing patterns in different parts of the thorax for the identification of possible COVID-19 infection.

**Approach:**

We used signal moment, signal texture, and shape moment features extracted from five different body regions of interest (whole upper body, chest, face, back, and side) of images obtained from thermal video clips in which optical flow and super-resolution were used. These features were classified into positive and negative COVID-19 using machine learning strategies.

**Results:**

COVID-19 detection for male models [receiver operating characteristic (ROC) area under the ROC curve (AUC) = 0.605 95% confidence intervals (CI) 0.58 to 0.64] is more reliable than for female models (ROC AUC = 0.577 95% CI 0.55 to 0.61). Overall, thermal imaging is not very sensitive nor specific in detecting COVID-19; the metrics were below 60% except for the chest view from males.

**Conclusions:**

We conclude that, although it may be possible to remotely identify some individuals affected by COVID-19, at this time, the diagnostic performance of current methods for body thermal imaging is not good enough to be used as a mass screening tool.

## 1 Introduction

Coronavirus disease-19 (COVID-19) is a respiratory disease caused by the coronavirus “severe acute respiratory syndrome-related coronavirus 2” (SARS-CoV-2)^1^ that was declared a pandemic by the World Health Organization (WHO) in March 2020. According to the WHO and as of the end of August 2021, the number of global cases reached over 216 million, and the number of confirmed deaths reached four and a half million.^2^ The respiratory illness may cause acute respiratory distress syndrome characterized by pulmonary infiltrates and hypoxemia, with dry cough, fever, and fatigue being the main symptoms.^3^^,^^4^

The main diagnostic tool for SARS-CoV-2 is a deoxyribonucleic acid test based on a polymerase chain reaction (PCR) assay,^3^^,^^5^^,^^6^ which requires respiratory specimens obtained by nasal or pharyngeal swabs.^5^ The results are typically delivered between 2 and 5 days after sampling. Other technologies have been explored in this regard; in particular, medical images from computed tomography (CT)^7^^,^^8^ have reported a prediction accuracy of 89% and an area under the receiving operating characteristic curve (ROC) of 0.92. These results suggest that imaging may be a useful tool to aid in the diagnosis of COVID-19. Nevertheless, CT uses ionizing radiation and requires unique installations along with a complicated process that limits the number of possible tests per equipment, and the economic costs can be prohibitively high for screening a large population.

Human body temperature has been used since the beginning of medicine as an indicator of health and disease. Today, modern infrared imaging systems offer high-resolution (HR) images able to detect small temperature changes.^9^ Body thermography can be a useful method to evaluate or investigate several clinical conditions that alter body temperature values and distribution.^10^ Abnormal thermal patterns are easily recognizable by infrared thermography (IRT) and can be used to establish correlations with diseases. Although this type of technique is imprecise and depends on the surrounding environment, IRT has some strong advantages: it is contactless, non-invasive, and devoid of harmful radiation effects; large areas can be monitored simultaneously; and it can be done in real time.^11^ In particular, its ability for mass screening can be highly beneficial during pandemic emergencies. According to Perpetuini et al.,^12^ the use of IRT is encouraged, particularly in healthcare and transport hubs, such as airports, because they are places where high numbers of possible infected people are expected to be found.

Artificial intelligence (AI) has been shown to improve thermography-based diagnosis in three main ways. First, these algorithms can reduce the workload of experts, so they can focus more on difficult cases. Second, AI reduces inter-observer variability as thermogram diagnosis can sometimes be subjective to human, so mathematics increases objectivity. Third, AI improves diagnosis quality, for diagnosis done by humans is heavily reliant on both their experience and their physical and mental state.^13^ Currently, there are AI applications already approved for clinical diagnosis.^14^ IRT has been previously used to support the detection of diseases such as breast cancer,^15^ aided by machine learning (ML), and to successfully support the diagnosis of other respiratory disorders^16^ without the aid of ML. To our knowledge, the potential of thermal imaging for identification of COVID-19 and specifically thermal videos has not been investigated enough. Martinez-Jimenez et al.^17^ conducted a study with 80 volunteers using IRTs to explore exclusively the face, they compared temperature values and temperature distribution on the face of healthy volunteers and patients with and without a COVID-19 infection. We think a more thorough examination of body thermal images needs to be conducted. Considering the previous studies, it may be possible that infrared videos could be used to support COVID-19 detection because SARS-CoV-2 infection in viremia stages is uniformly characterized by changes in body temperature and breathing patterns.^18^ Particular respiratory patterns were analyzed in individuals that tested positive for COVID-19, in contrast to healthy individuals and individuals with asthma, and they were found to be distinguishable (AUC of above 80%).^19^ In addition, studies show that pulmonary and laryngeal involvements in people with COVID-19 can cause insufficient airflow that affects breathing and voice regularity.^20^^,^^21^ Thus, in principle, it is important to investigate the potential of video recording of body temperatures to support the identification of suspected individuals in an early stage of the disease. The hypothesis was that the small differences in skin temperature could be used as a fiducial marker that can be tracked and characterized, and hence, it may provide a practical method for tracking breathing patterns, for breathing patterns and thermal imaging have been correlated in previous studies.^22^^–^^27^ Hence this paper presents the evaluation of the role of thermal imaging, specifically thermal video, for the identification of SARS-CoV-2 infected people using AI. Furthermore, we explore the role of breathing patterns in different parts of the upper body for the identification of a possible SARS-CoV-2 infection.

## 2 Methodology

### 2.1 Thermal Dataset

**Fig. 1:**
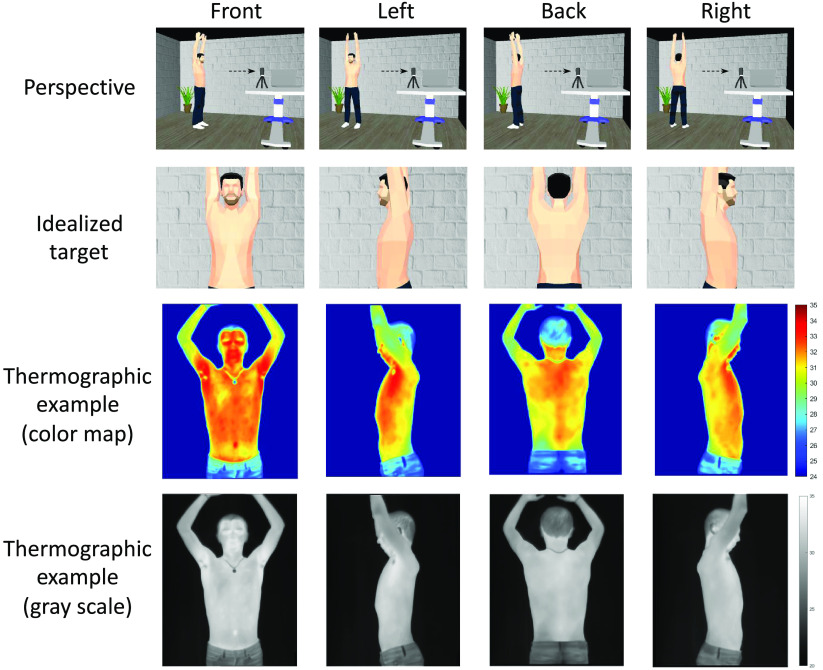
Thermal imaging session. A single continuous take of thermal video was done to capture 10 s of patients breathing and 10 s holding their breath.

A total of 252 volunteers were enrolled in an Institutional Review Board approved prospective study aimed at testing the ability of thermal videos to detect SARS-CoV-2. The study protocol was approved by the Ethics and Research Committees at Escuela de Medicina y Ciencias de la Salud, Tecnológico de Monterrey. The participants’ ages ranged from 18 to 75 years. The number of participants per gender with their height, weight, and PCR results are summarized in Table 1. The study recorded a set of measurements from participants regarding PCR-results, demographics, vital signs, participant activities, medications, respiratory symptoms, and a thermal video session in which the volunteers performed, in a secluded cabin, simple breath-holds during the video capturing in four different positions—front, back, left, and right—as seen in Fig. 1. The room temperature was maintained in the vicinity of 25°C, and among the 55 participants registered as having fever, the highest body temperature registered was 37.7°C; therefore, it was not expected that the presence of droplets of sweat could exert any influence on the recording. Thermal images were recorded in video mode, mostly at five frames per second, using a Digital Thermal Imaging Camera TI-128 from Omega Engineering Inc. (800 Connecticut Ave. Suite 5N01, Norwalk, CT 06854). The camera was connected via a USB to a laptop computer running a Windows^®^ operative system from Microsoft^®^ as suggested by the provider instructions. The acquisition software Omega TI Analyzer version 4.1.8.6875 was used. Drivers and acquisition software were obtained and installed following provider instructions. Table 2 shows the patient demographics and vital signs at the time of video capture; significant differences were assessed using a t-test. Table 3 shows the patient symptoms and medical conditions; significant differences were calculated with a comparison of frequencies using a chi-square test. Regarding the medical conditions, 11 subjects presented diabetes and 15 presented arterial hypertension. In cases of diabetes and arterial hypertension, it is expected that the entities may have disordered sleep breathing.^28^^,^^29^ However, in the case of hypertension, only the pulmonary arterial hypertension was previously correlated to this disorder; therefore, we do not expect breathing anomalies during the video capture due to this condition.

**Table 1:** Number of enrolled subjects per sex and PCR results. A total of 251 subjects (after removing the sole participant with PCR results that were not reported) were considered for further analyses.

PCR result	Female	Male	Total
Positive	21	38	59
Negative	72	120	192
Not reported	1	0	1
Total	94	158	***252***

**Table 2:** Characteristics of the enrolled subjects stratified by sex and PCR result. Significant differences between positive and negative results are highlighted.

Demographics and vital sigs	Males			Females		
	Positive	Negative	AUC	Positive	Negative	AUC
Age, years	37.92 (11.6)	37.39 (37.4)	0.53	34.00 (11.9)	36.64 (14.3)	0.55
Weight, kg	87.87 (16.8)	84.61 (84.6)	0.53	65.60 (13.8)	67.30 (15.7)	0.52
Height, cm	175.21 (6.9)	173.63 (173.6)	0.56	160.38 (7.3)	162.06 (6.2)	0.58
Cardiac rate (beats/min)	**84.90 (16.4)** a	77.18 (77.2)	0.65	78.25 (11.6)	81.10 (12.4)	0.55
Systolic pressure, torr	**129.50 (13.3**)^b^	132.33 (132.3)	0.56	118.50 (11.6)	120.96 (15.9)	0.53
Diastolic pressure, torr	85.00 (11.2)	83.53 (83.5)	0.48	79.65 (5.7)	77.78 (9.0)	0.58
Temperature, forehead, °C	36.82 (0.7)	36.61 (36.6)	0.59	36.68 (0.5)	36.66 (0.5)	0.50
Oxygen saturation (${\mathrm{SpO}}_{2}$)	0.96 (0.0)	0.96 (1.0)	0.52	**0.96 (0.0)** b	0.97 (0.0)	0.58

a p<0.001

b p<0.05

**Table 3:** Symptoms and conditions of the enrolled subjects. Significant differences between positive and negative results are highlighted.

Condition or symptoms	Males			Females		
	Positive (No|Yes)	Negative (No|Yes)	AUC	Positive (No|Yes)	Negative (No|Yes)	AUC
Cough	**25|14** a	106|13	0.63	**8|12** b	61|12	0.72
Headache	**16|23** b	88|31	0.67	**7|13** c	46|27	0.64
Anosmia	**25|14** b	113|6	0.65	**14|6** c	64|9	0.59
Ageusia	**25|14** b	113|6	0.65	16|4	66|7	0.55
Fever	**24|15** d	100|19	0.61	11|9^d^	60|13	0.64
Fever medication	**29|10** b	117|2	0.62	17|3	69|4	0.55
Sore throat	**21|18** d	93|26	0.62	**9|11** c	50|23	0.62
Analgesics	**29|10** d	110|9	0.59	**15|5** c	67|6	0.58
Muscle pain	**25|14** d	100|19	0.60	**12|8** c	58|15	0.60
Joint pain	**32|7** c	110|9	0.55	**13|7** d	65|8	0.62
Malaise	**29|10** c	105|14	0.57	15|5	63|10	0.56
Chills	35|4	112|7	0.52	16|4	64|9	0.54
Vomit	38|1	118|1	0.51	20|0	71|2	0.51
Diarrhea	34|5	106|13	0.51	16|4	64|9	0.54
Hypertension	36|3	112|7	0.51	19|1	70|3	0.50
Diabetes	37|2	114|5	0.51	19|1	69|4	0.50

a p<0.001

b p<0.0001

c p<0.05

d p<0.01

### 2.2 Video Preprocessing

The video acquisition protocol included breathing in four positions, as shown in Fig. 1. Each thermal video was visually inspected to determine all of the frames for these body positions. Each infrared spectrograph video segment was then converted into a standardized black-and-white MPEG-4 video with gray levels that were standardized between 62°F and 102°F. Therefore, four MPEG-4 were generated and labeled with front, left, back, and right corresponding to each body position.

**Fig. 2:**
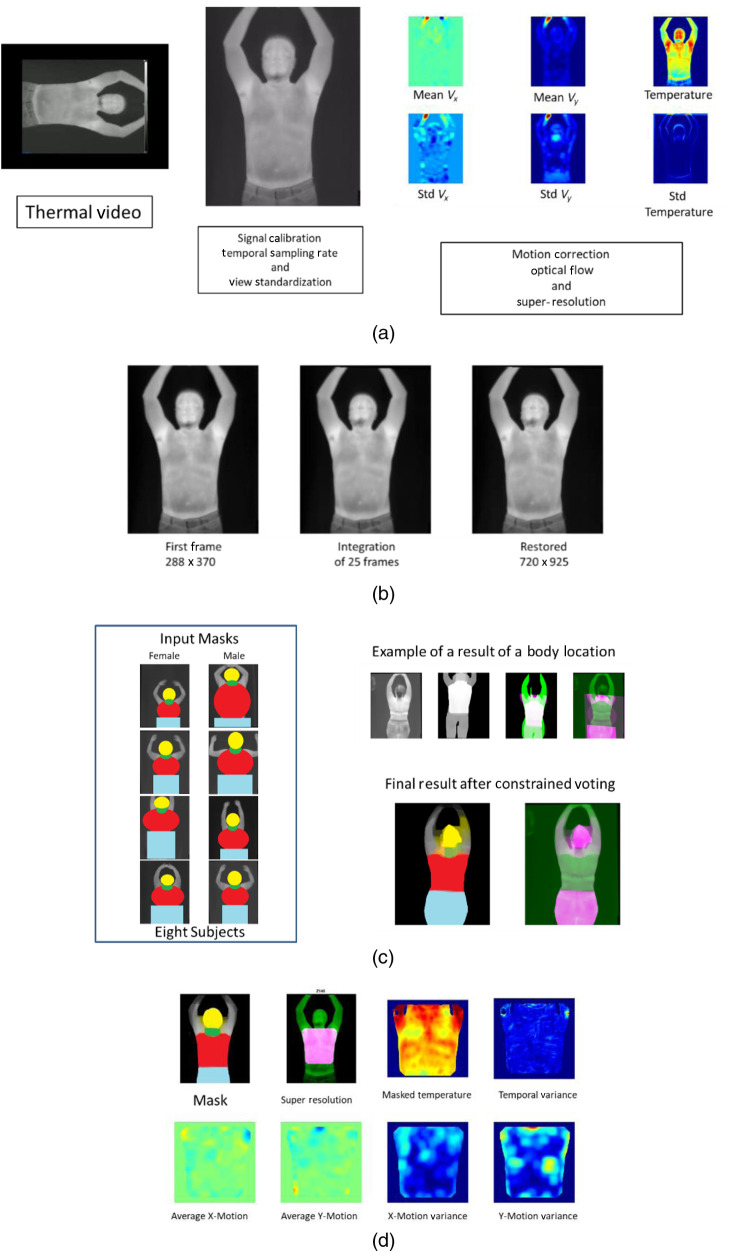
(a) Processing: a thermal video is oriented, calibrated, framerate standardized, and converted into a set of six images. (b) The first 25 frames of each video are integrated into an HR image. (c) Segmentation process: eight labeled HR images are used to vote for the definitions of the human body regions of interest (ROIs). (d) ROI quantitation: each ROI is used to mask a specific body area to be quantitated.

After that, all four MPEG-4 video clips were standardized and corrected for sampling differences, temperature-calibration variations, blur, and large motions. Then, the Lucas Kanade optical flow method was used to estimate the frame by frame skin motion for each one of the videos.^30^ Motion correction based on rigid transformations was used to integrate the first 25 frames of each video clip and generate an HR thermal image.^31^ After optical flow and super resolution, a set of six images were generated. The first image was the HR thermal image. The second image consisted of the pixel-by-pixel temperature variance observed during the breath-hold. The third and fourth images consisted of the average optical flow in the horizontal and vertical directions. The last two images were created by estimating the frame-wise variance of the optical flow. In summary, the derived image set consisted of two temperature-driven images and four motion-derived (optical flow) images. Figure 2 shows the video processing steps used for this research work.

### 2.3 Image Segmentation and Body Regions of Interest Definitions

Image segmentation and regions of interest (ROI) definition were done using two different approaches. The first approach of image segmenting based on the Otsu method identified the entire upper body and removed background information.^32^ The first segmentation process was applied to each of the six images separately, and all segmented images were reviewed and manually refined to avoid loss of information in missed segmented body regions due to the presence of hair, necklaces, masks, and underwear.

The second approach for image analysis was the identification of the ROI. The identification of the head, chest, and back was done using an atlas-based approach.^33^^,^^34^ HR images from eight subjects (four males and four females) were manually labeled with head, chest, neck, and legs. Figure 2 shows an example of the labeled images used for the atlas-based segmentation approach. Once all subjects were labeled, an affine registration method was used to match each one of the atlas subjects with the HR thermal image for each subject. Once we have the eight segmentations per subject, we use the majority-vote approach to get the final label of each pixel. The majority-vote segmentation was then refined by a morphological closing operation that corrected segmentation errors in the neck and breast regions. All video preprocessing and image segmentation was done using MATLAB. (2010). version 7.10.0 (R2010a). (The MathWorks Inc., Natick, Massachusetts)

### 2.4 Image Feature Extraction

Heat and motion patterns were quantified by extracting image features from the global segmentation and the extracted ROIs. From the global segmentation-based approach, we extracted textural descriptors and signal moments from all pixels inside the segmentation mask and for each one of the six images at three different image resolutions. The textural features quantified the spatial signal heterogeneity using the gray level co-occurrence matrix (GLCM) and local binary patterns (LBP). Details about the mathematical definitions of textural features can be found in previous literature.^35^^,^^36^ Descriptive statistics of the signal distribution inside the segmentation were also collected using the moment features. Finally, fractal dimension formulation was used to characterize the association of the scale dependent features to changes in resolution

$$D_{i}=\frac{1}{m}\sum_{n=1}^{m}(\begin{array}{c} \log(\Delta F_{i})/\log(\Delta r)\\ k \end{array}),$$ (1)

where $F_{i}$ is a scale dependent feature, $\Delta F_{i}$ is the change in feature for each Δr change in resolution (r=1/scale) k is the number of acquired scales, and m is the total number of possible combinations. Table 4 summarizes the extracted descriptors.

**Table 4:** Summary of the features extracted from each of the six summary images [Fig. 2(a)] after segmentation.

Group	Target	Description
Moment	Global segmentation and ROI	Basic statistics of signal distribution. Descriptors included: mean, mass, area, standard deviation, skewness, kurtosis, quantile location [p = {0.01, 0.05, 0.25, 0.5, 0.75, 0.95, 0.99}], entropy, and coefficient of variation.
GLCM	Global segmentation and ROI	Texture features at different resolutions: three for Global, and four for ROI depicting the degree of correlation between pair pixels in different aspects: contrast, dissimilarity, homogeneity, angular second moment, energy, and correlation.
LBP	ROI	Texture features depicting the association between the central pixel and its neighbors. They were computed using five neighbors and four radii = {1, 2, 3, 5}. And reporting LBP(r=5), and the Max {LBP(r=5) – LBP(r=1), LBP(r=3) – LBP(r=1), LBP(r=2) – LBP(r=1)}
Fractal	ROI	Equation (1) applied to change in the surface signal area, and changes of GLCM features at the four different resolutions: {r=1,2,4,8}

### 2.5 Data Conditioning, Features Transformation, and Demographic Adjustments

**Fig. 3:**
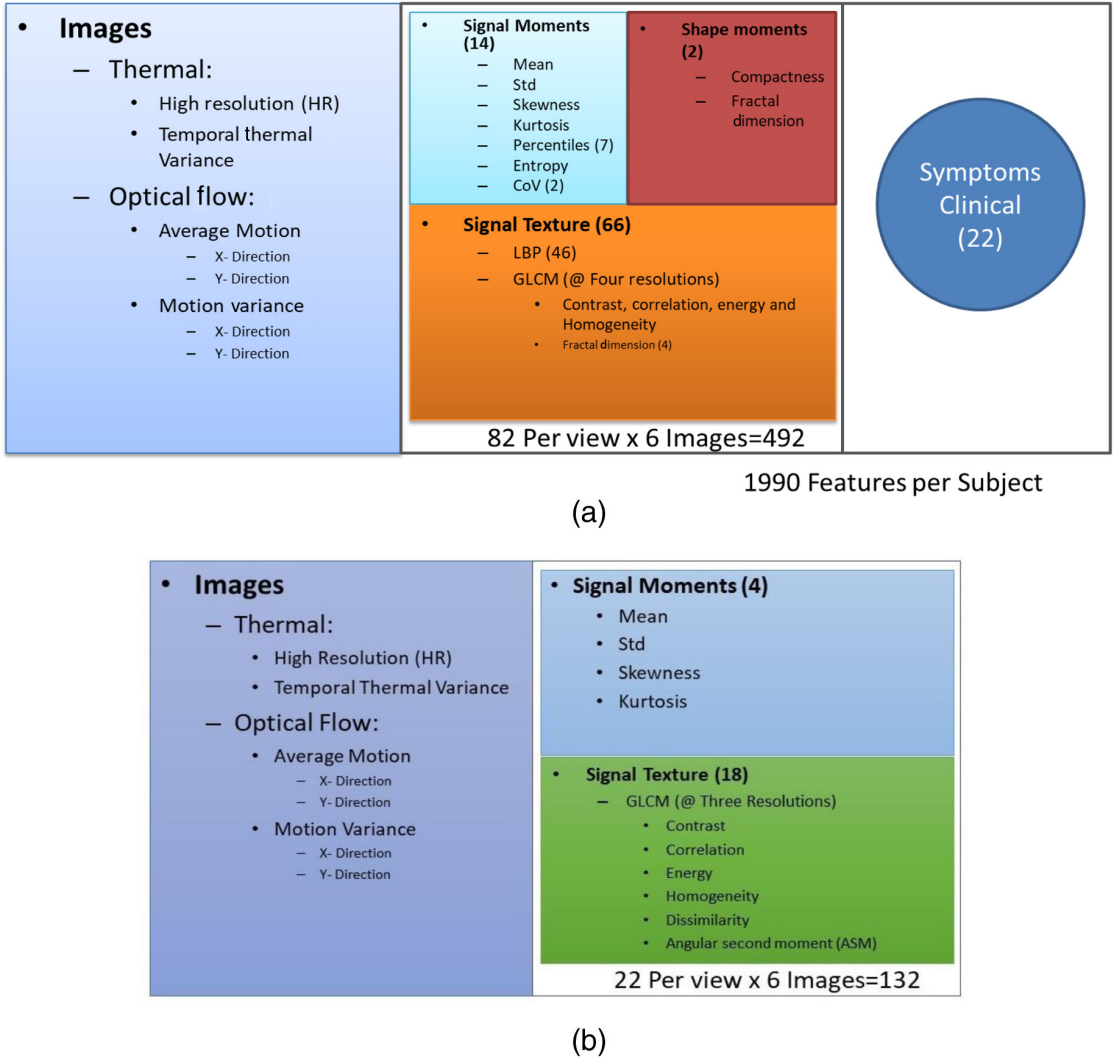
Summary of the features extracted on (a) each ROI per view for a total of 1990 features for a specific subject and (b) the global segmentation for a total of 132 when using all views together.

Figures 3(a) and 3(b) show the set of features used for each ROI and for the global segmentation, respectively. Regarding Fig. 2(a), the 1990 features extracted per subject were obtained considering 82 features (14 signal moments, two shape moments, and 66 signal texture) from each of the six images and each of the four views (chest, face, back, and side) and the 22 symptoms and clinical data (82×6×4+22=1990). Missing data were imputed using the nearest neighborhood approach.^37^ Regarding data conditioning, the power transformation, Yeo-Johnson transformation, or pseudo log transformation was applied to highly skewed features. To mitigate the effect of age, gender, height, and size of ROI, all image features of the training set were adjusted using a robust fitting model from the FRESA.CAD R package version 3.3.0. For adjustment purposes, asymptomatic or subjects with only one symptom that were not taking any temperature modifying medication (non-steroidal anti-inflammatory drugs) were considered to be reference control. Once adjusted, all features were decorrelated selecting a set of basis vectors using the feature decorrelation function of FRESA.CAD.^38^ Finally, all transformed, adjusted, and decorrelated features were z normalized.

### 2.6 Machine Learning

To ascertain the role of thermal imaging and ML in COVID-19 diagnosis, we explored the data set using two different approaches. First, we explored a small set of ML methods using the whole-body segmentation to research the impact of ML methods in COVID-19 assessment using demographics, vital signs, symptoms, and thermal image data. Second, we tested the hypothesis that different parts of the body have different powers in detecting COVID-19 from thermal videos with and without the aid of vital signs and symptoms.

For the first data exploration, we split the data into 70% training and 30% testing sets, and we considered five classifiers: support vector machine (SVM), AdaBoost, Random Forest, Naïve Bayes, and K-nearest neighbors (KNN).^39^^–^^43^ 200 repetitions of cross-validation were applied.

Image segmentation, feature extraction, and transformation processes were done using Python programming language (2019) version 3.7.6 (Python Software Foundation^44^). Adjustment and classification were executed using FRESA.CAD in R Core Team (2020) version 4.0.2. (R: A language and environment for statistical computing, R Foundation for Statistical Computing, Vienna, Austria^45^).

**Fig. 4:**
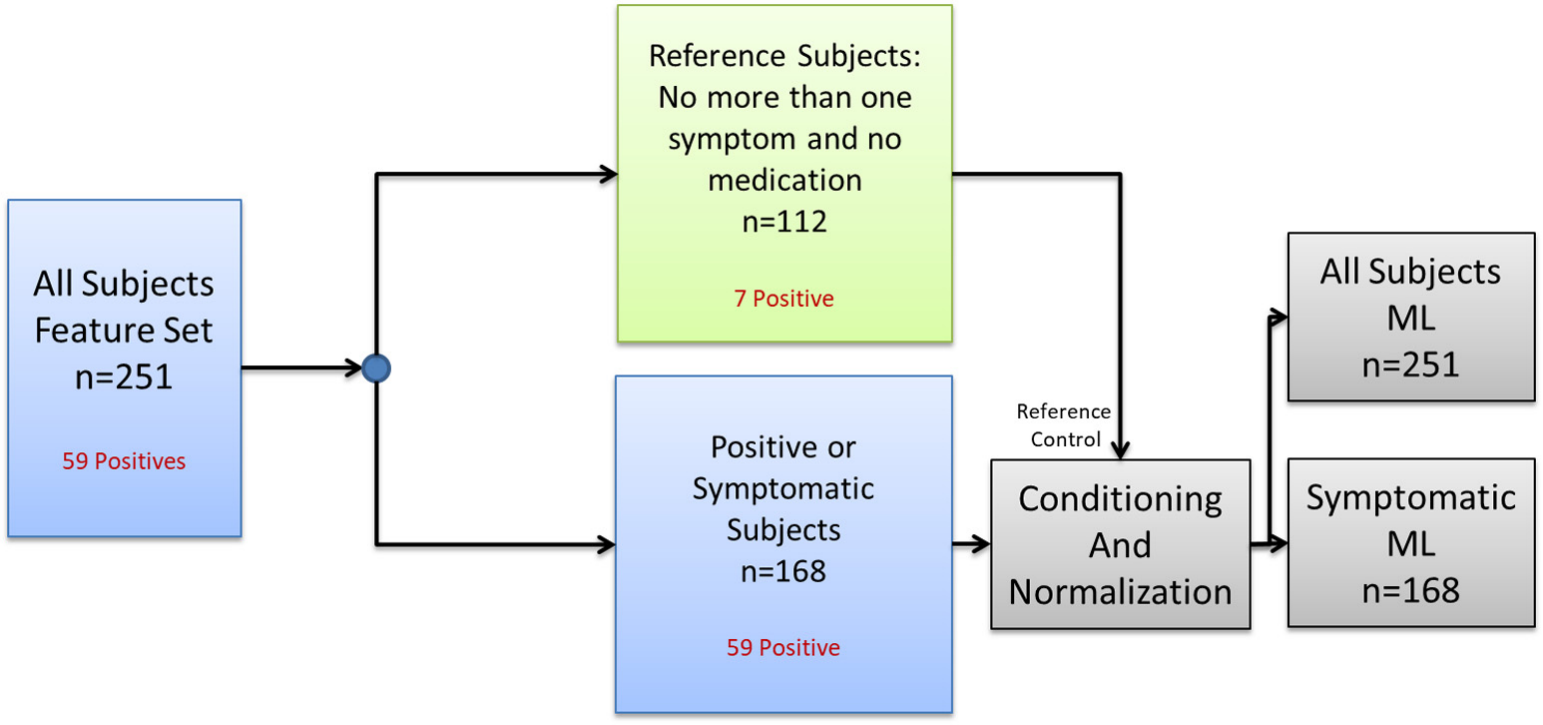
Splitting of the subjects for conditioning and normalization before fighting the ML model.

For the second set of experiments, the subjects were split as in Fig. 4. We selected Naïve Bayes as the ML classifiers, and we varied the ROI used for the detection of COVID-19, as well as the set of features to be selected for COVID-19 prediction under two scenarios. The first scenario used all subjects (symptomatic and asymptomatic), for the second scenario, only symptomatic subjects were used for training and validation. The analyzed ROIs were chest, face, back, and the combination of left and right views. For this set of experiments 500 leave two out cross-validations without class balance were used to document the performance of COVID-19 detection.

### 2.7 Statistical Analysis

All of the test results of the ML experiments were aggregated for consensus prediction. The consensus predictions (median of all test-results per subject) were analyzed for balanced error rate (BER):

BER=1−0.5×(sensitivity+specificity), (2)

where the sensitivity=(correct positive)/N, specificity=(correct negative)/N, and N is the total number of sampled subjects. The overall accuracy (ACC=(total correct)/N) was also reported for the ML comparison experiments. The continuous probability of positive COVID-19 was described using the receiver operating characteristic (ROC) plots and reporting the area under the curve (AUC). Models were created for all individual views (left, right, front, and back) as well as for the entire set. Analysis of the model performance was created for the entire set, males, and females. The top selected features of the models were described and visualized as heatmaps; these features were obtained with the frequency of the selection in the repetitions during the cross-validation process.

## 3 Results

### 3.1 Global Segmentation Experiments

#### 3.1.1 Classification of four views

In this first task, classifications were performed with AdaBoost using the four views separately, including the 132 features extracted from the six thermal images [Fig. 3(b)] per subject, ending with a sample of 252. Classification results are summarized in Table 5. Accuracies of the four views are in the range of 0.433 (95% CI 0.37 to 0.50) to 0.524 (95% CI 0.46 to 0.59).

**Table 5:** COVID-19 classification performance using the four views separately.

Metric	Back		Front		Left		Right	
	95% CI		95% CI		95% CI		95% CI	
Accuracy	**0.524**	**(0.46, 0.59)**	0.433	(0.37, 0.50)	0.409	(0.38, 0.47)	0.496	(0.43, 0.56)
AUC	**0.515** b	**(0.43, 0.60)**	0.435	(0.35, 0.52)	0.397	(0.31, 0.49)	0.470	(0.38, 0.56)
Sensitivity	0.475	(0.34, 0.61)	0.424	(0.30, 0.56)	0.373	(0.25, 0.51)	**0.492**	**(0.36, 0.63)**
Specificity	**0.539**	**(0.47, 0.61)**	0.435	(0.36, 0.51)	0.420	(0.35, 0.49)	0.497	(0.43, 0.57)
Balanced error	**0.493** b	**(0.43, 0.57)**	0.571	(0.50, 0.64)	0.604	(0.53, 0.67)	0.508	(0.44, 0.58)

a The best results per metric are given in bold.

b Significative results.

#### 3.1.2 Classification of all views, examined with five classifiers

In this experiment, we explored the classification with a larger sample using the thermal features including the four concatenated views; in this case we ended with a sample of 1008 (252 subjects × 4 four views). We examined the results using the five methods stated above (Sec. 2.6) to verify that the performance is not influenced by the classifier. Classification results are summarized in Table 6. All of the methods obtained classification accuracies in the range of 0.536 (95% CI 0.50 to 0.57) to 0.569 (95% CI 0.54 to 0.60).

**Table 6:** COVID-19 classification performance using all views together and five different classifiers.

Metric	SVM		AdaBoost		Random forest		KNN		Naïve Bayes	
	95% CI		95% CI		95% CI		95% CI		95% CI	
Accuracy	**0.569**	**(0.54, 0.60)**	0.555	(0.52, 0.59)	0.537	(0.51, 0.57)	0.536	(0.50, 0.57)	0.549	(0.52, 0.58)
AUC	0.567	(0.52, 0.61)	**0.572** b	**(0.53, 0.61)**	0.554	(0.51, 0.60)	0.557	(0.52, 0.60)	0.563	(0.52, 0.61)
Sensitivity	0.530	(0.46, 0.60)	0.534	(0.47, 0.60)	0.555	(0.49, 0.62)	0.576	(0.51, 0.64)	**0.580**	**(0.51, 0.64)**
Specificity	**0.582**	**(0.55, 0.62)**	0.549	(0.51, 0.59)	0.522	(0.49, 0.56)	0.521	(0.49, 0.56)	0.543	(0.51, 0.58)
Balanced error rate	0.448	(0.41, 0.48)	**0.438** b	**(0.40, 0.48)**	0.447	(0.42, 0.49)	0.449	(0.41, 0.49)	0.446	(0.41, 0.48)

a Te best results per metric are given in bold.

b Significative results.

#### 3.1.3 Female and male classification

**Fig. 5:**
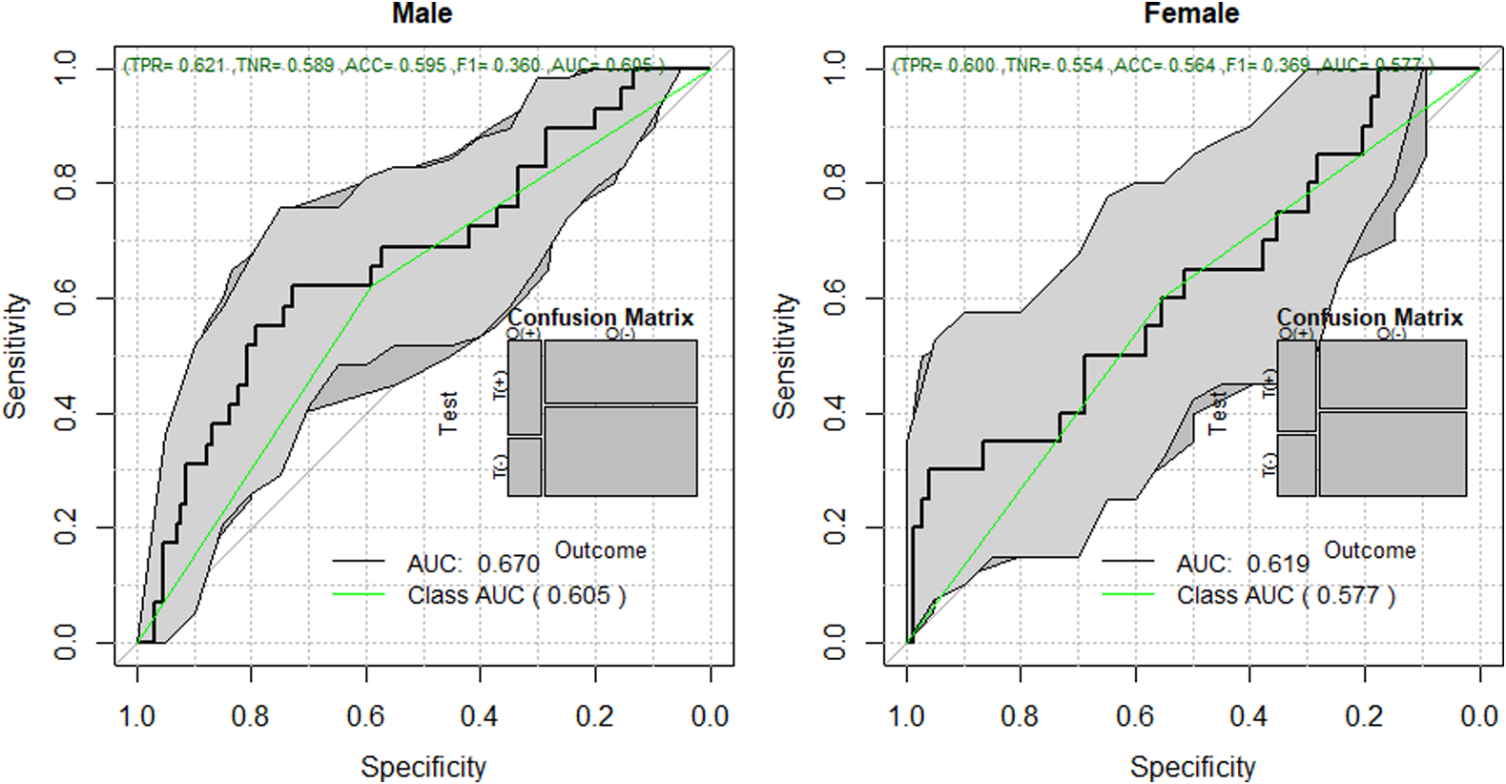
ROC for COVID-19 classification, comparison among female and male patients.

As in the last classification experiments, here we used the 132 thermal features with a sample of 251 (Table 1) subjects stratified by sex, with the not reported subject being removed, and ending with a sample of 93 for the female classification and 158 for the male classification. We used AdaBoost to compare results among female and male patients of individual views. Results from the front view are reported here, for outcomes from different views did not present significative differences. Although both performances are almost similar, male patients tended to be classified with slightly better accuracy than females (Fig. 5).

#### 3.1.4 Classification including vital signs and symptoms

**Fig. 6:**
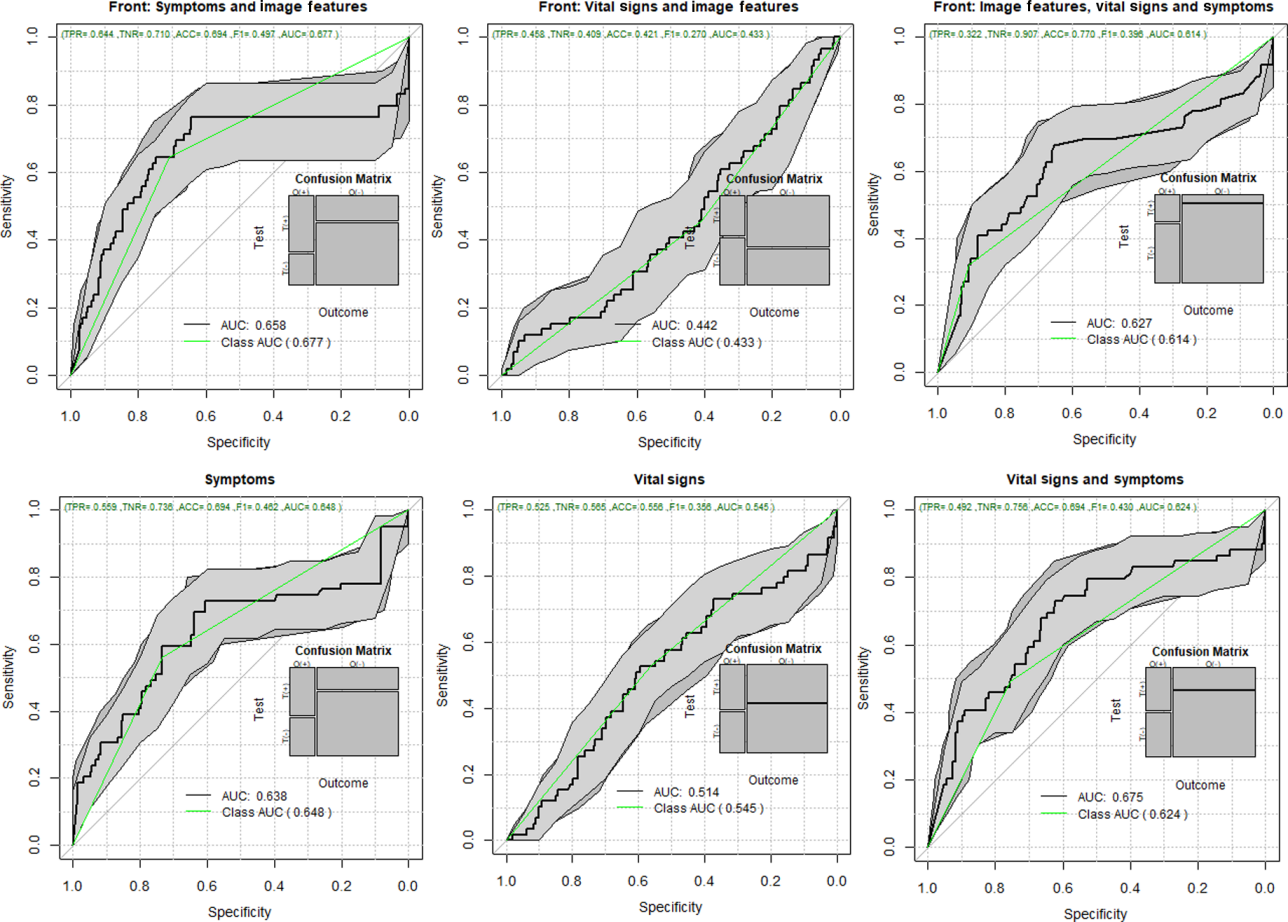
ROC for COVID-19 classification using thermal features, vital signs, and symptoms.

Vital signs, such as temperature (°C), systolic, diastolic, heart rate, and oxygen saturation; symptoms, such as sore throat, diarrhea, vomit, anosmia, ageusia, shivering, headache, myalgia, and arthralgia; and the total number of symptoms were also included for classification. Similar to the previous experiment, the results reported here were obtained by taking features extracted from the front view. The resulting areas under the curve are shown in Fig. 6. We show results when including image features in three different cases: coupled with symptoms—142 features (132 thermal features and 10 symptoms), with vital signs—137 features (132 thermal features and 5 vital signs), and with symptoms and vital signs together—147 features (132 thermal features, five vital signs, and 10 symptoms). We also present baselines, without thermal features, in the same three cases for comparison.

### 3.2 Regions of Interest Experiments

The segmentation results of the ROI-based experiments were visually inspected to avoid including in the analysis segmentation results that failed to correctly identify the correct body component or that had other issues affecting the quality of the thermal data, such as beard, face mask, and large motion artifacts. The quality inspection indicated that one subject from the chest ROI had to be excluded. A total of 18 subjects were removed from the face ROI, 15 from the back ROI, and five from the left-right ROI analysis.

**Fig. 7:**
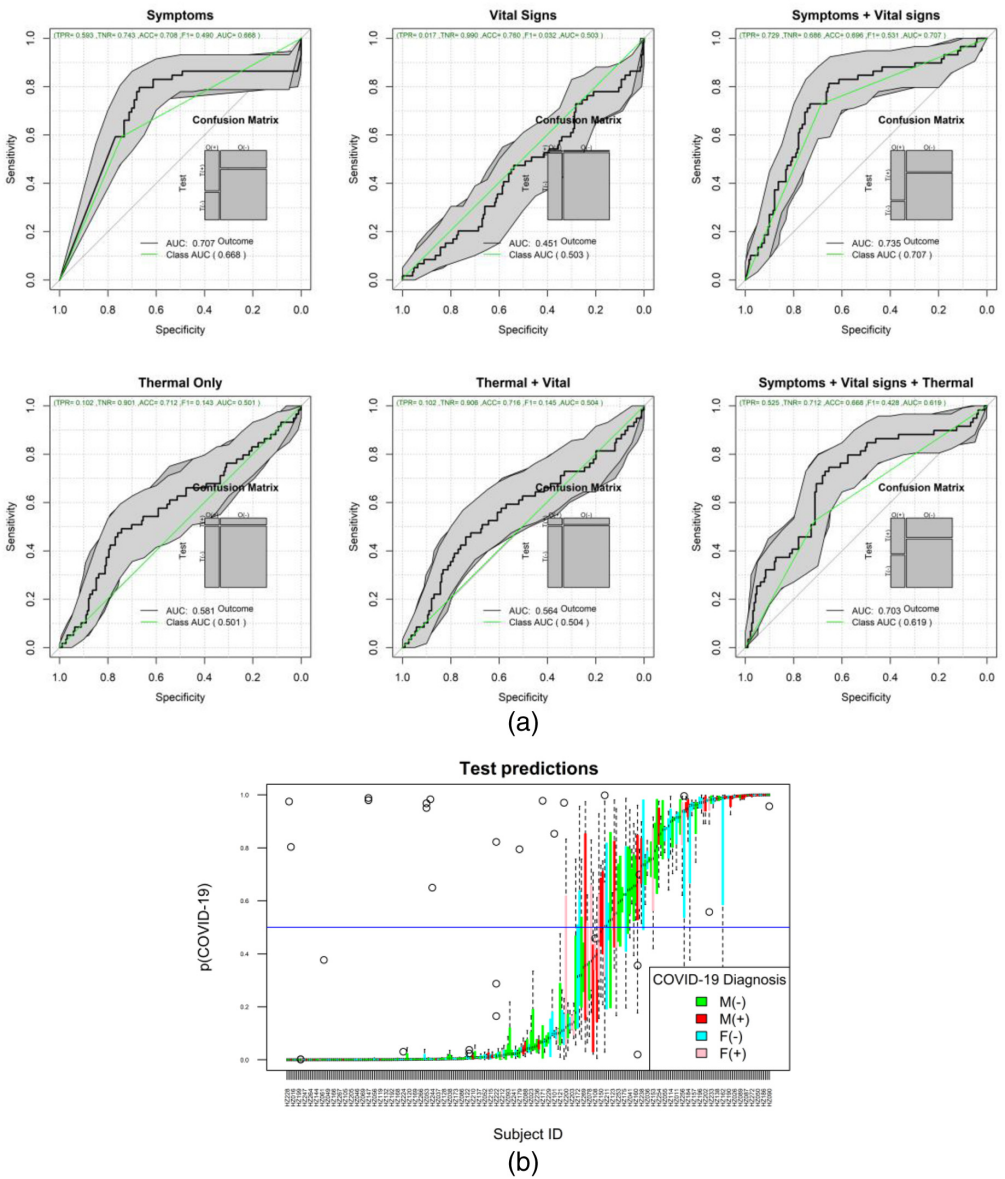
(a) ROC of the different feature combinations. (b) Predictions of each one of the 500 repetitions per subject for the all-feature model.

**Fig. 8:**
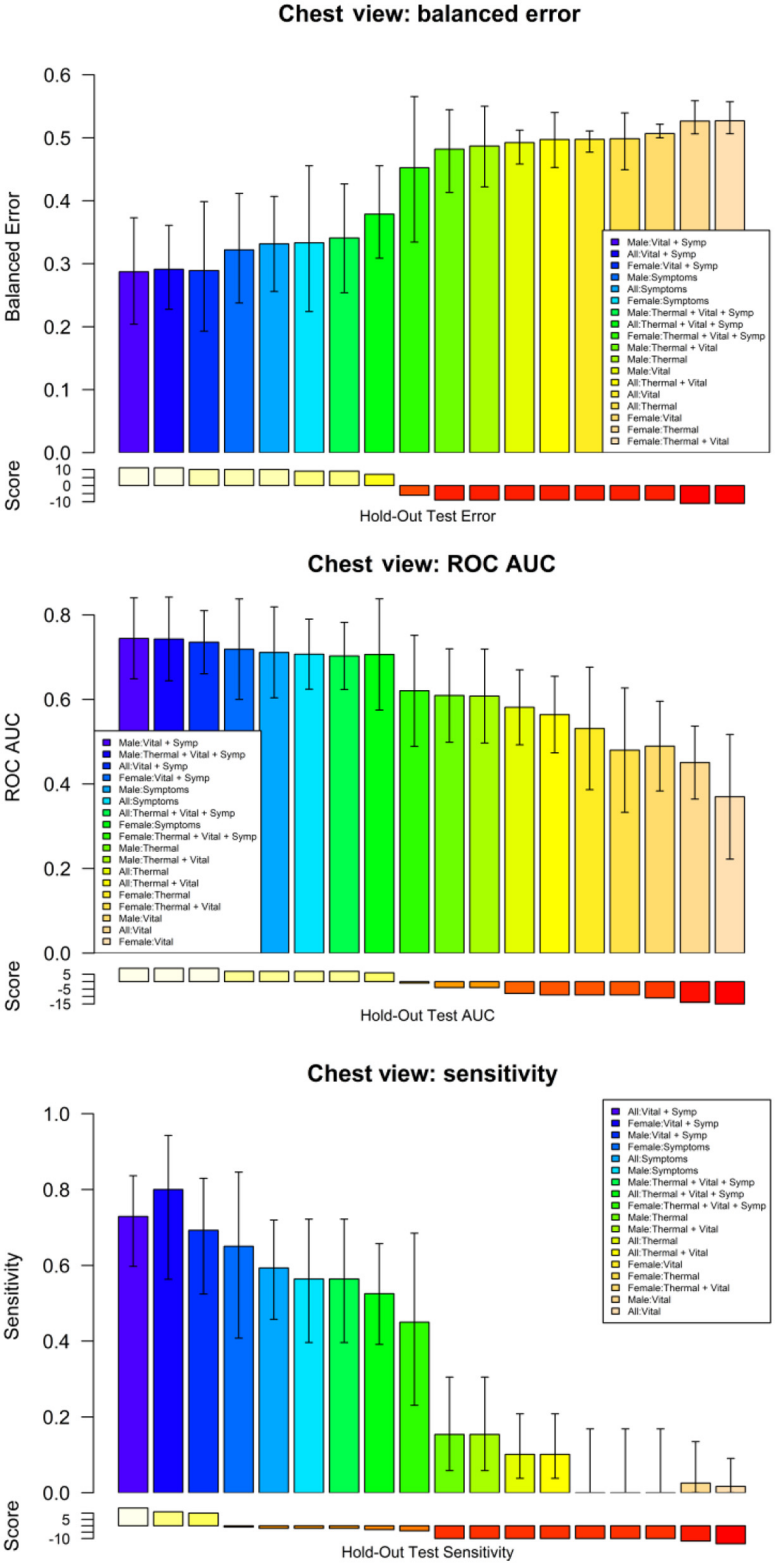
Comparison of key metrics across all models for the different feature combinations and stratified by sex.

The ML results of the second set of experiments are shown in Figs. 7 and 8 for the chest ROI. Figure 7(a) shows the ROC analysis for the combinations of features set: symptoms, vital-signs, and thermal features. Figure 7(b) shows the boxplot of the testing results distribution for each subject and sorted by the predicted probability of COVID-19. Figure 8 shows a detailed comparison of the ML models for the different feature combinations and data stratification: all, males, and females.

**Fig. 9:**
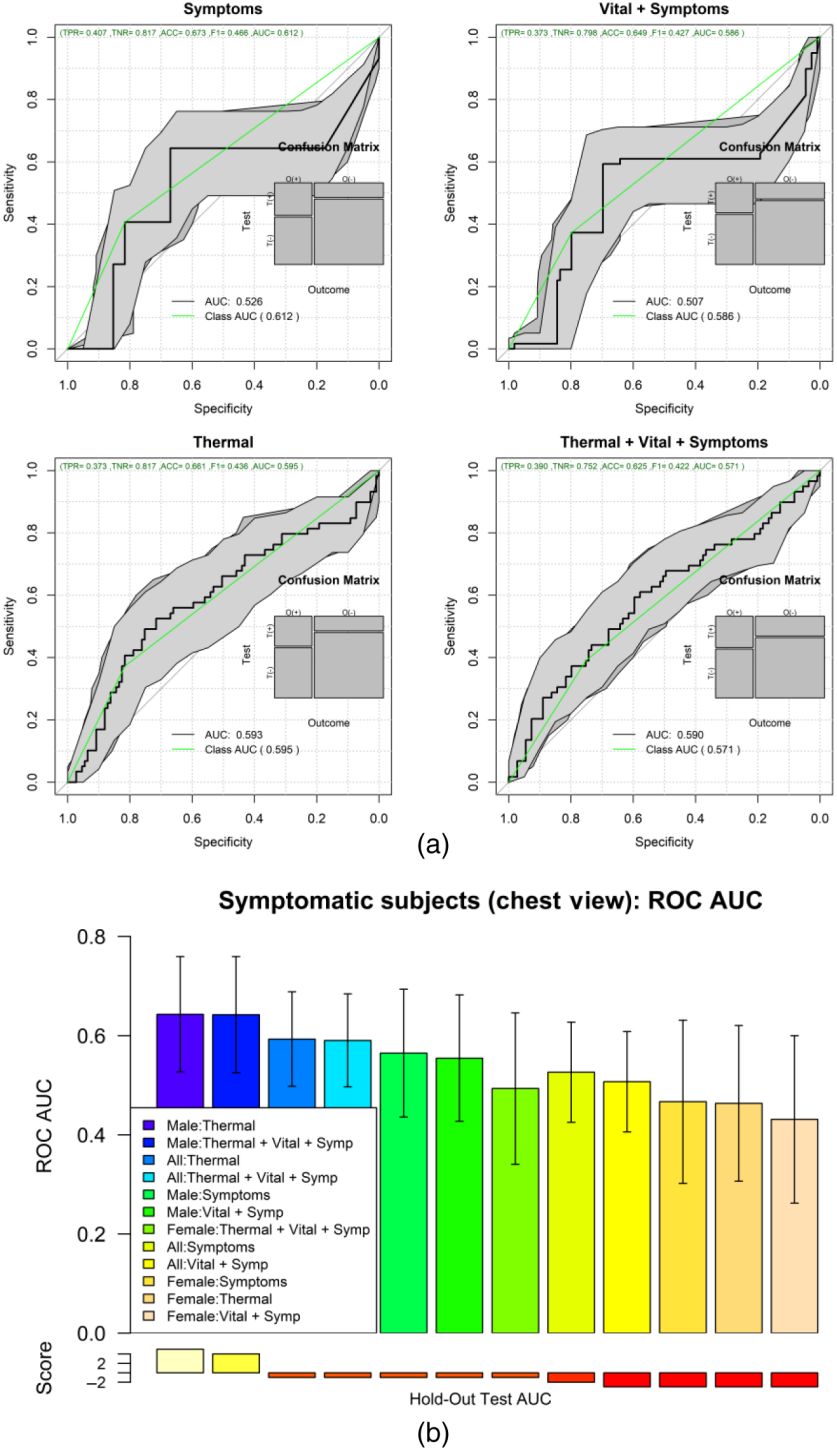
(a) ROC analysis of only symptomatic subjects, for combinations of symptoms and thermal imaging data. (b) Comparison of the AUC for the different combinations of view and stratified by sex.

**Fig. 10:**
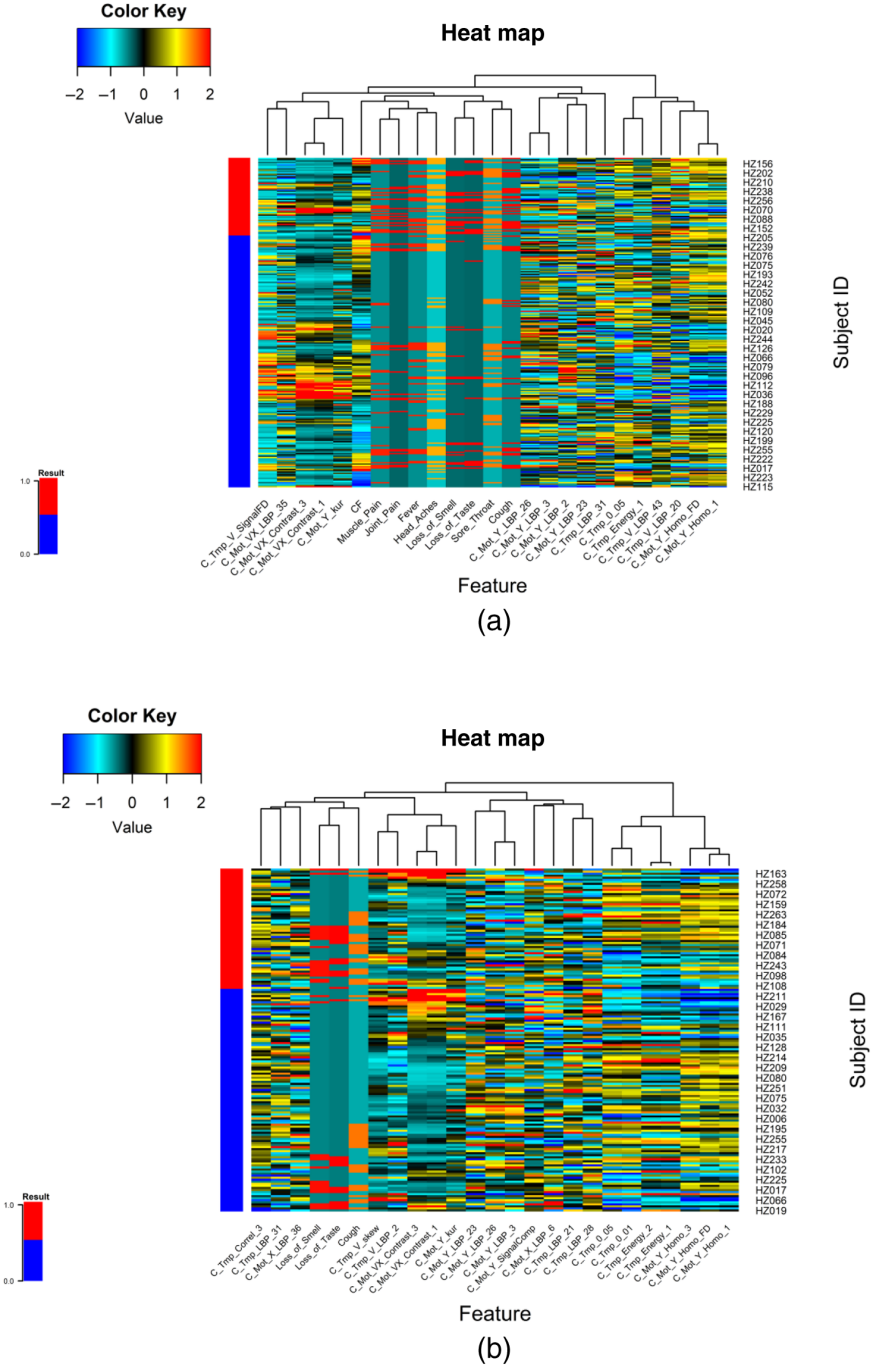
Heatmaps of the top selected features associated with the presence of COVID-19. (a) Features for symptomatic and asymptomatic subjects. (b) Top features that separate COVID-19 positive and negative results on the set of symptomatic subjects.

Figure 9(a) shows the ROC analysis of the symptomatic only subjects from the chest ROI; Fig. 9(b) shows the box plots of the 500 test results per subject, ordered by the predicted COVID-19 probability. Figure 10(a) shows the heatmap of the top features associated with COVID-19 when using all subjects. Figure 10(b) shows the heatmap of the features associated with COVID-19 when using only symptomatic subjects. Supplementary material in Appendix A shows the detailed results of the ROI experiments for chest, back, left-right, and face experiments.

## 4 Discussion

The thermal COVID-19 study is the first comprehensive study of the potential role of thermal imaging in the remote detection of respiratory issues due to the presence of SARS-CoV-2 infection. We were able to recruit 251 (59 COVID-19 positive) patients with complete demographics, vital signs, symptoms, and thermal video acquisitions showing breathing patterns at four different views of each volunteer. The data are publicly available for interested researchers that may want to explore image analysis algorithms or ML algorithms for the detection of respiratory illnesses.^46^^,^^47^ The vital signs and clinical data acquired in this study suggest that body temperature is not an important discriminant for COVID-19 classification, confirming the results of published studies. Further, we observed that cardiac rate (beats/min) was higher in positive males than healthy counterparts (CR = 84.9 versus 77.2; p<0.001). It is worth noticing that we confirmed that COVID-19 symptoms are slightly different in symptomatic subjects with anosmia (loss of smell) and ageusia (loss of taste).

Regarding the role of thermal imaging, this paper presents a comprehensive evaluation using ML methods via the exploration of thermal and breathing patterns and observations of the breathing process at four different views. Furthermore, we explored the role of these patterns in five different body ROIs: whole upper body, chest, face, back, and side views (left and right). We present the exploration of thermal features using five different ML strategies for the classification of positive and negative COVID-19. The results indicate that even the best performing ML method, AdaBoost, may be insufficient in COVID detection with an ROC AUC of 0.572 with 95% CI from 0.53 to 0.61 and that COVID-19 detection in male models (ROC AUC = 0.605 95% CI 0.58 to 0.64) is more reliable than in female models (ROC AUC = 0.577 95% CI 0.55 to 0.61). The results of the ROI study indicate that thermal imaging is not very sensitive nor specific in detecting COVID-19. Regarding the ROI of the whole upper body, the classification performance tends to improve slightly in Fig. 6, when using symptoms coupled with thermal features (ROC AUC improvement = 22.3%) and when using all information together (ROC AUC improvement = 19.2%) compared with the performance of only thermal features of the front view (Table 5). However, when looking at the performance when using only symptoms, and only symptoms and vital signs, we can conclude that the direct contribution of the thermal images is not as significative as the medical information (ROC AUC improvement when thermal features are added to symptoms = 2%; ROC AUC improvement when thermal features are added to vital signs and symptoms = −4.8%). Similar behavior is found when inspecting the chest ROI (Fig. 7); the performance of vital signs and symptoms (ROC AUC = 0.73) does not improve when thermal features are added (ROC AUC = 0.703). The strongest response was obtained from features extracted from the chest ROI analysis of males, as shown in Figs. 8 and 9 (ROC AUC = 0.64 95%CI = 0.53 to 0.76), and thermal data from the face had a role in female subjects where the ROC AUC was 0.60 95%CI = 0.50 to 0.69. Regarding important thermal and dynamic features of COVID-19, the data of the chest area indicated that variations in the vertical motion patterns from males are significative (Fig. S3 in the Supplemental Material). This finding confirms that breathing patterns are different between positive and negative COVID-19 patients. On the other hand, temperature-related features were selected among important features in the face, back, and left and right analysis. The skewness of the temperature distribution in females was highlighted as an important feature of positive COVID-19. The fact that differences in temperature distributions are distinct in COVID-19 symptomatic subjects may shed light on some of the aspects of how COVID-19 infections differ from other respiratory illnesses. We must point out that the study did show differences between males and females, but these differences may be due to the smaller sample size of the female population that also failed to show any statistical difference in vital signs when compared with the male subgroup.

The study is not free from limitations. Although we tried to register the best possible thermal images, some subjects were out of focus, some moved during the acquisition period, and the camera error is expected to have some degree of interference in the results, namely, measurement accuracy of the camera corresponds to ±2°C or ±2% @ environment temperature 10°C to 35°C, while among the participants the temperature variance during the video capture was <1.75°C and 1.4°C for negative and positive participants, respectively, with the median around 0.55°C in both cases (Fig. S8 in the Supplemental Material). These acquisition artifacts introduce noise in the already small sample size for this proof-of-concept study. Two other limitations of the study are the large imbalance between males and females and the fact that all female volunteers were using bras that varied in size and shape, which affected all chest ROI analysis. Furthermore, many males had beards or mustaches, which affected comparisons in face ROI analysis.

Future work on the role of thermal imaging may be directed toward the estimation of true motion patterns via the analysis of three-dimensional (3D) estimated motion, instead of the use of optical flow method. Furthermore, considering the fact that we acquired video at fully 3D body rotation, it may be possible that other body motion patterns or temperature variations from start to end of the imaging session may be used to document associations to COVID-19 or other medical conditions.

## 5 Conclusion

This paper presents an evaluation of the possible role of thermal imaging and motion analysis for the remote diagnosis of COVID-19. This study is a more complete version than a preliminary study using the same dataset.^48^ The results indicate that it may be possible to remotely identify some males and some females affected by COVID-19; however, at this point, the performance of the methods used in this work as well as the thermal camera are not good enough to be used as a mass screening tool, although it may be useful in getting a better understanding of the role of thermal imaging as an aid in the differential diagnosis tool in medical conditions.

## 6 Appendix A: Supplemental Material

The results of all views are provided in the Supplemental Material.

## Code, Data, and Materials Availability

The subset of clinical and image data can be accessed via (https://doi.org/10.13026/pgk4-gx55). Researchers interested in the full data set must sign a disclosure agreement and make the request to the corresponding author.
