# Supplementary material for: COVID-19 classification using thermal images
Source: J Biomed Opt. 2022 May 18;27(5):056003. doi: 10.1117/1.JBO.27.5.056003 (PMC9116467; doi:10.1117/1.JBO.27.5.056003)
Supplement: Supplementary file 1 [file JBO_027_056003_SD001.pdf]

Appendix A: Supplemental Material

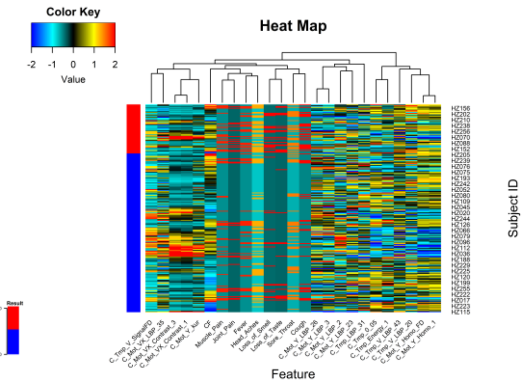

(a)

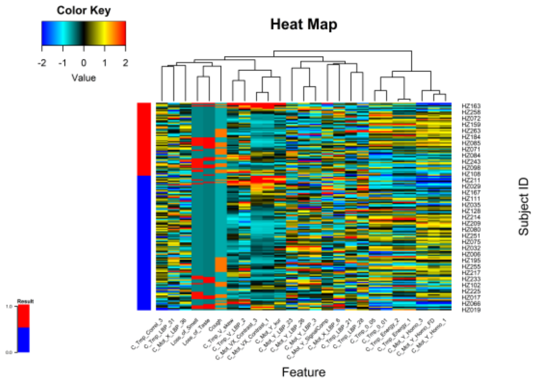

Chest

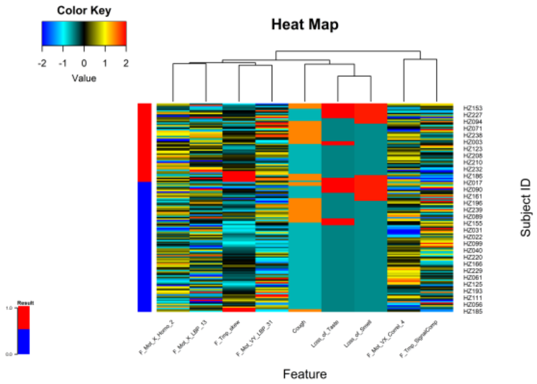

Face

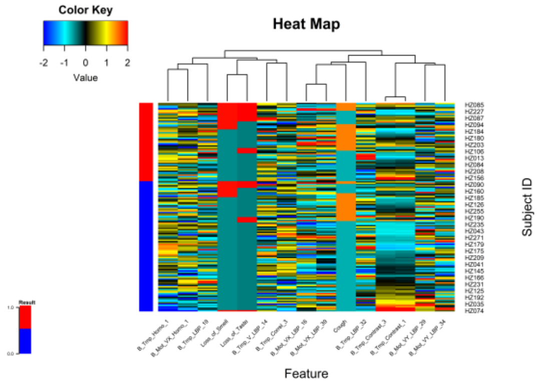

Back

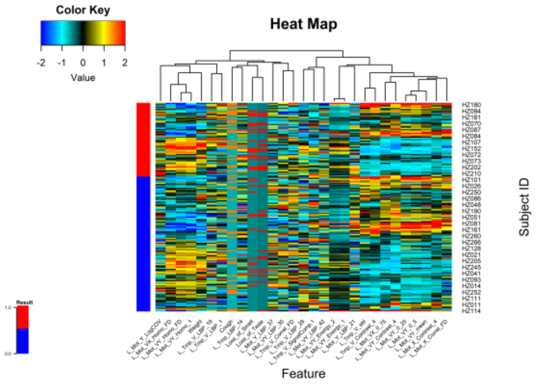

Left-Right

Fig. 1 Heat maps of top selected features for the chest view analysis. (a) Chest ROI: All subjects. (b) Only symptomatic subjects.

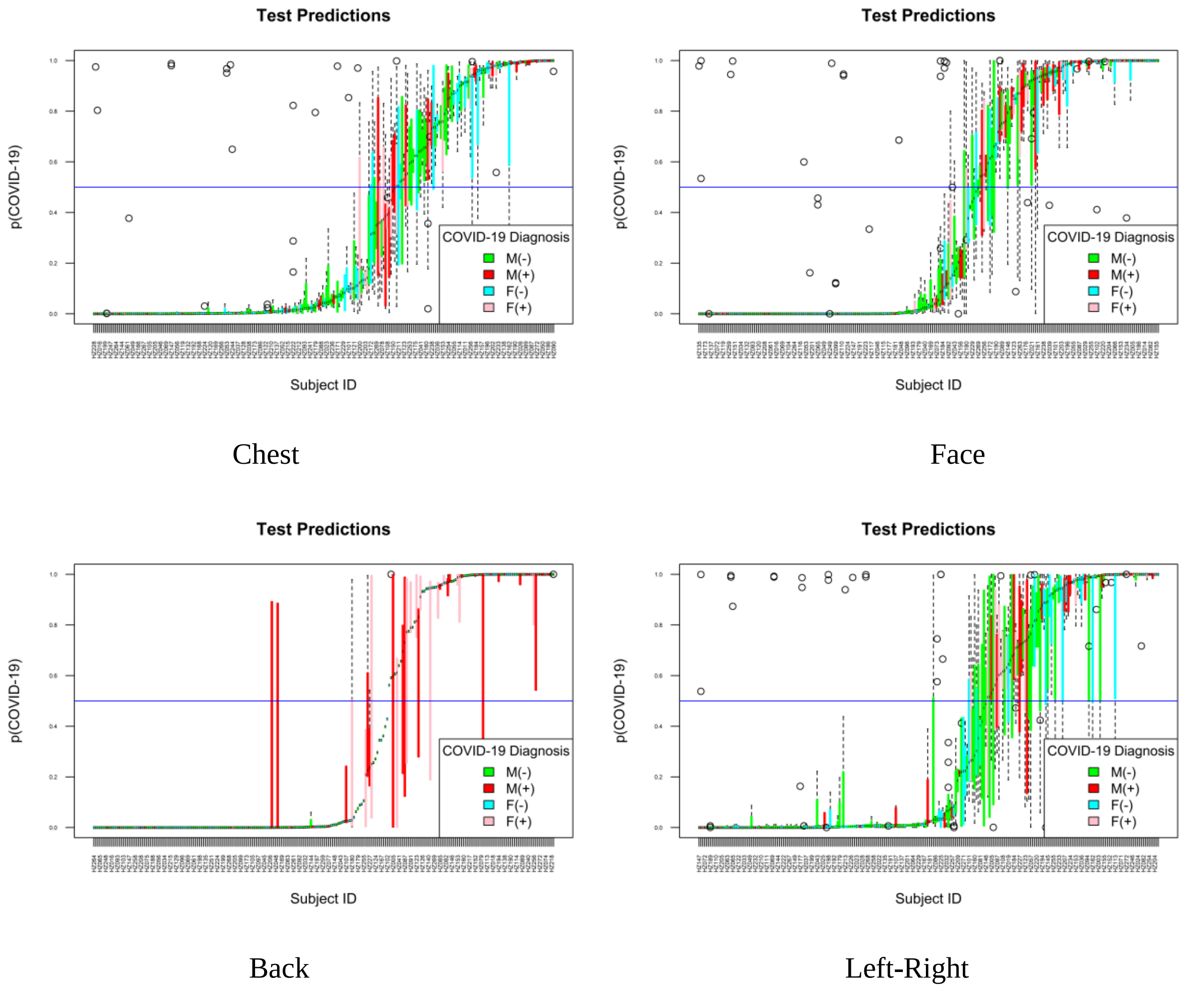

Fig. 2 Test results of each one of the 500 repetitoin of the Random CrossValidation for the four explored body ROIs

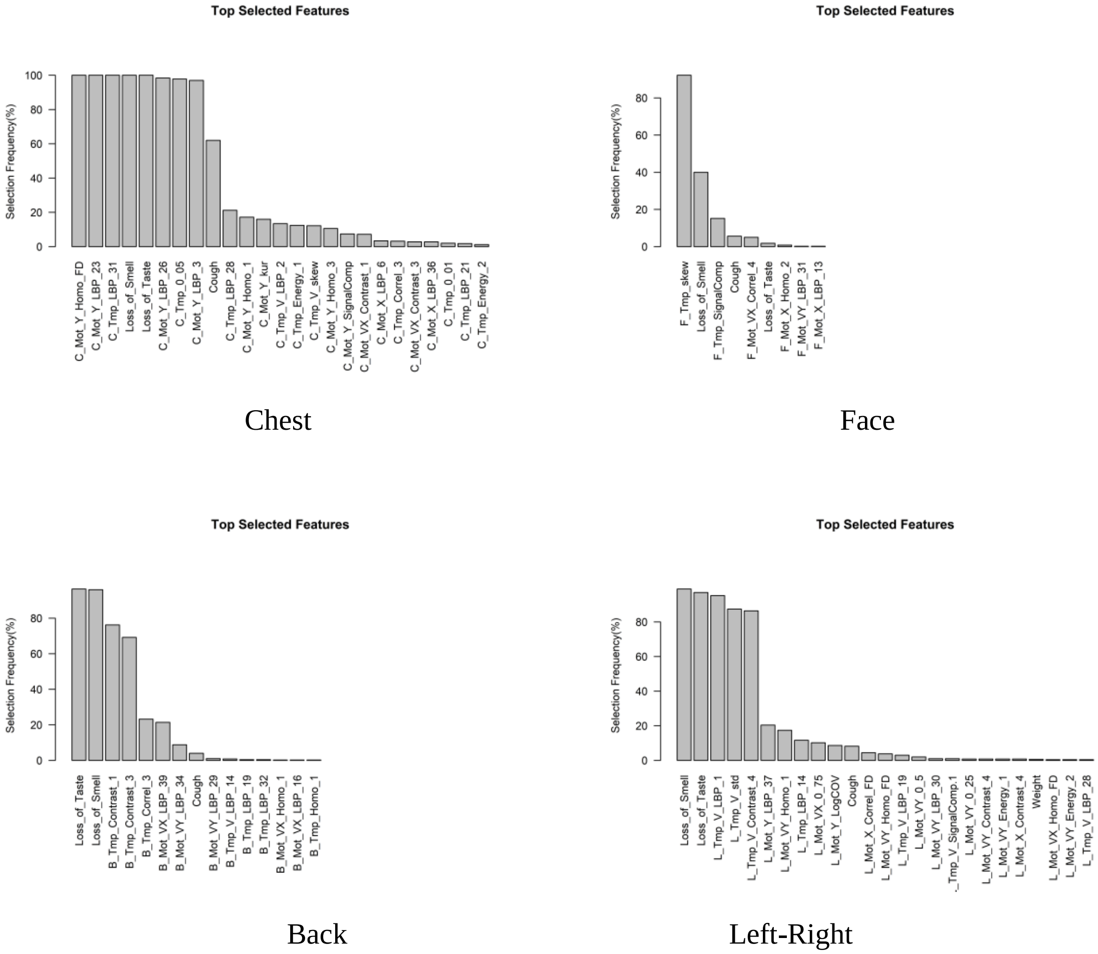

Fig. 3 Selection Frequency of Thermal Features at the different Body ROI

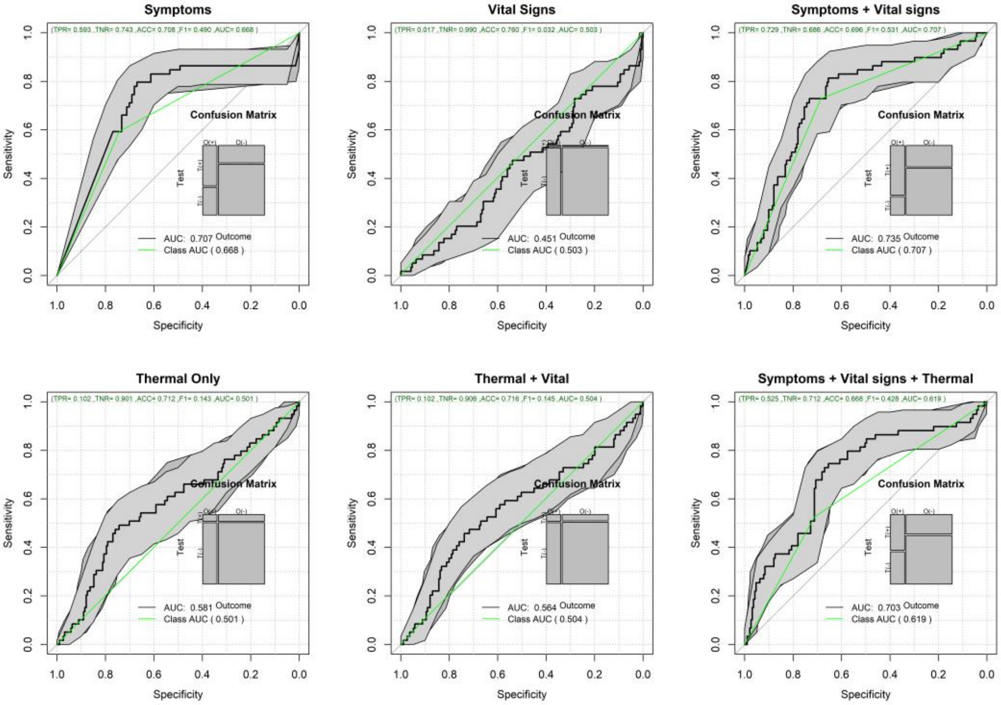

(a)

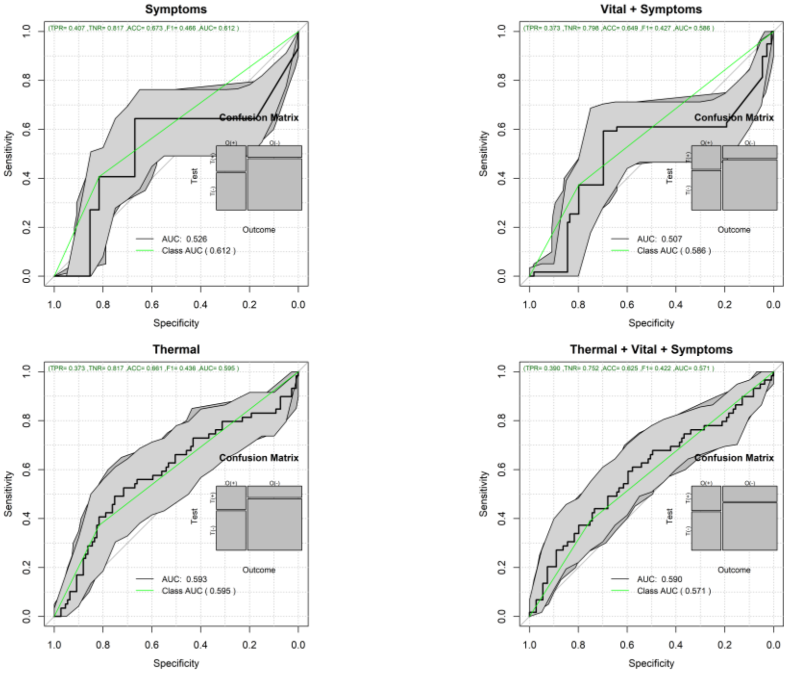

(b)

Fig. 4 ROC plots of the chest view using male and female subjects. (a) All subject data. (b) Only symptomatic subjects

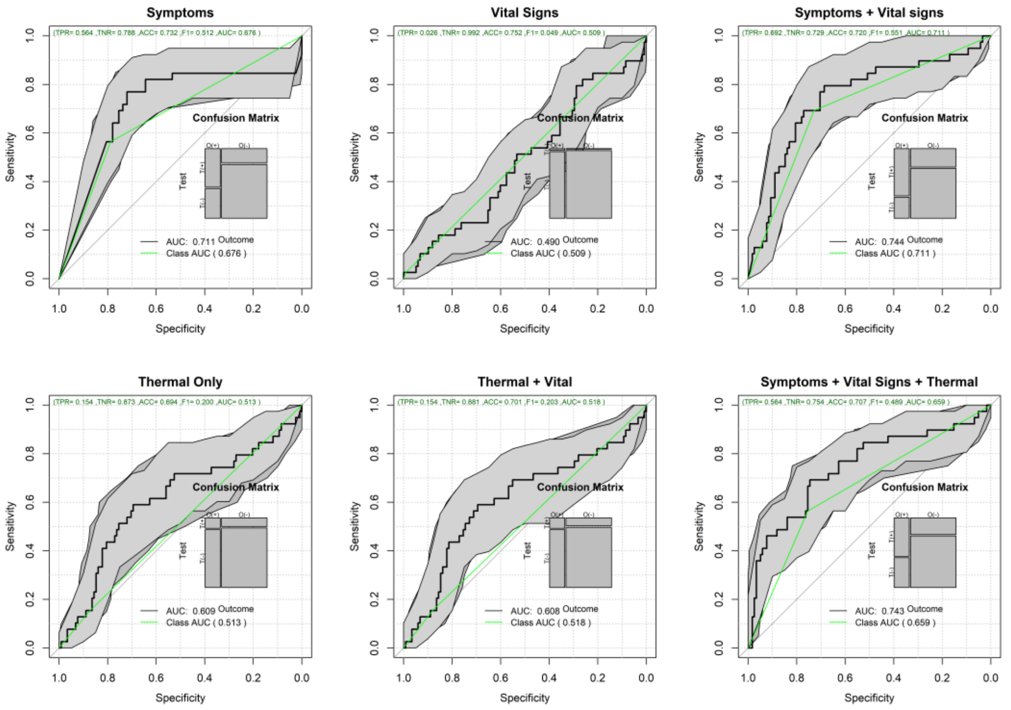

(a)

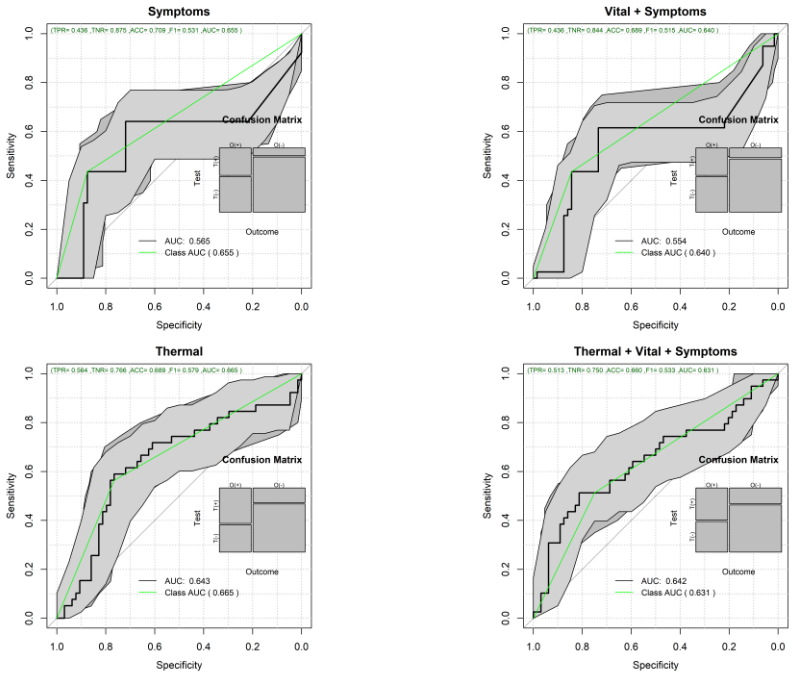

(b)

Fig. 5 ROC plots of the chest view form males. (a) All subject data. (b) Only symptomatic subjects

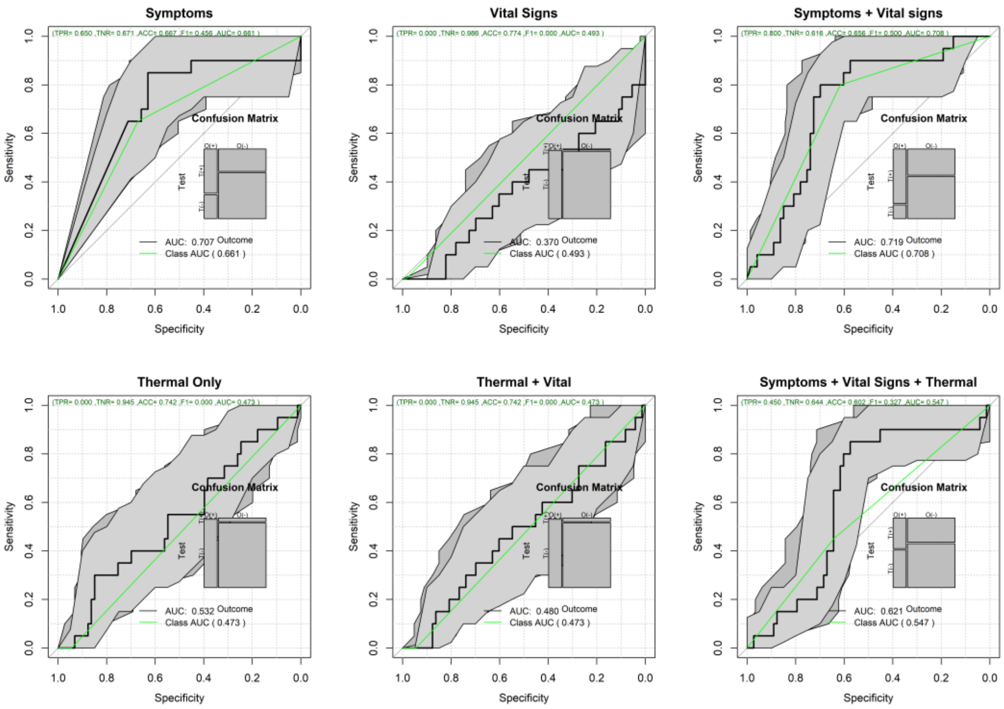

(a)

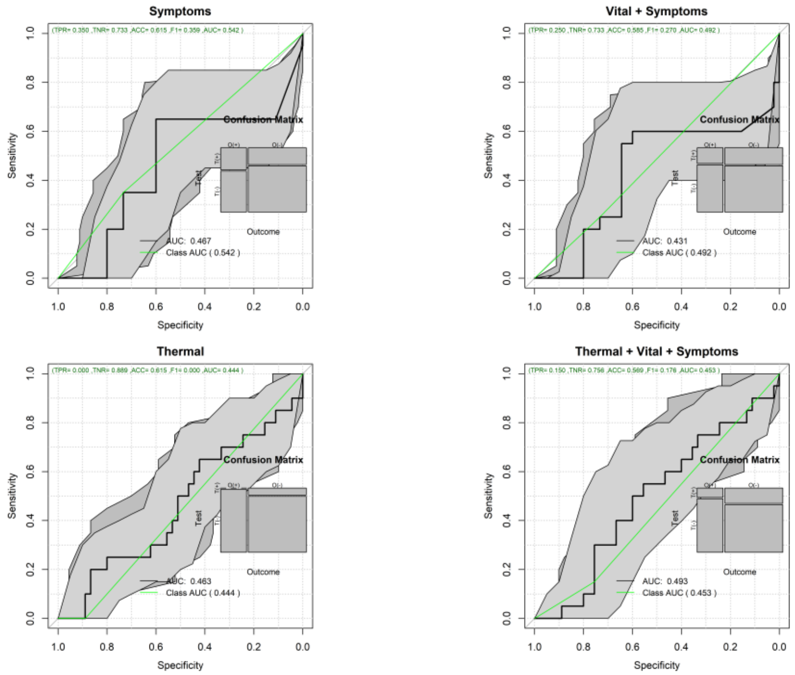

(b)

Fig. 6 ROC plots of the chest view for female subjects. (a) All subject data. (b) Only symptomatic subjects

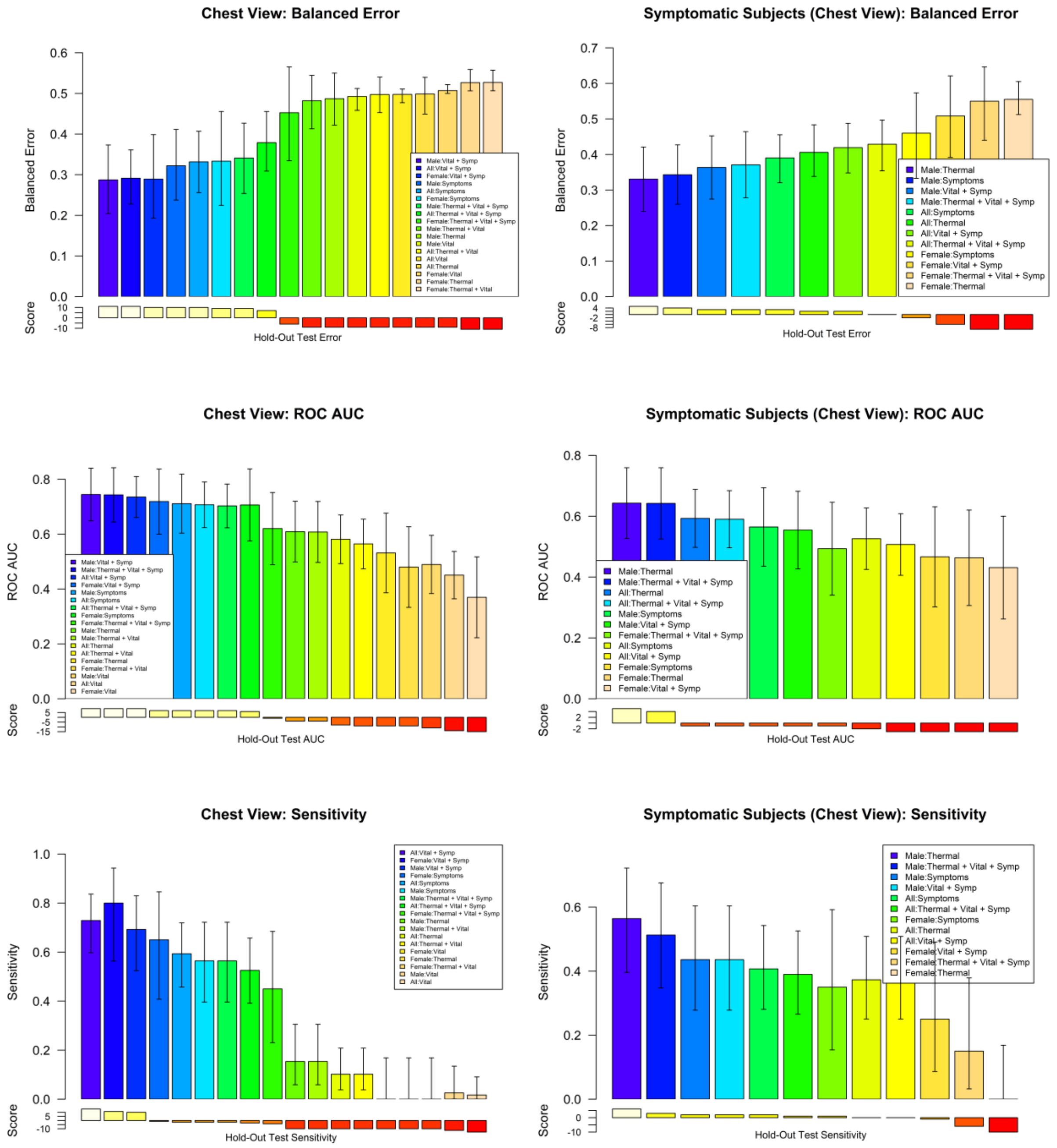

Fig. 7 Comparisons of key metrics for all subjects and symptomatic subjects of the chest view.

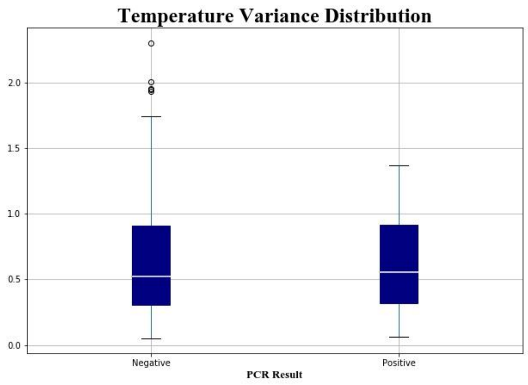

Fig. 8 Temperature variance distribution participants during the video capturing stratified by PCR results.

Table 1 Balanced error rate (BER) for all subject analysis

BER		Thermal			
	No Imaging	Chest	Face	Back	Left-Right
**All:Symptoms**	**0.332 (0.26-0.41)**				
**All:Vital**	0.498 (0.48-0.51)				
**All:Vital + Symp**	**0.291 (0.23-0.36)**				
**All:Thermal**		0.499 (0.45-0.54)	0.512 (0.49-0.53)	0.518 (0.49-0.54)	0.535 (0.52-0.55)
**All:Thermal + Vital**		0.497 (0.45-0.54)	0.512 (0.49-0.53)	0.518 (0.49-0.54)	0.532 (0.52-0.55)
**All:Thermal + Vital + Symp**		**0.379 (0.31-0.46)**	**0.310 (0.24-0.39)**	**0.310 (0.24-0.38)**	**0.300 (0.24-0.37)**
**Male:Symptoms**	**0.322 (0.24-0.41)**				
**Male:Vital**	0.492 (0.46-0.51)				
**Male:Vital + Symp**	**0.287 (0.20-0.37)**				
**Male:Thermal**		0.487 (0.42-0.55)	0.515 (0.48-0.54)	0.522 (0.48-0.56)	0.555 (0.53-0.59)
**Male:Thermal + Vital**		0.482 (0.41-0.54)	0.514 (0.48-0.54)	0.518 (0.48-0.55)	0.550 (0.53-0.58)

**Male:Thermal + Vital + Symp**		**0.341 (0.25-0.43)**	**0.293 (0.21-0.38)**	**0.315 (0.23-0.40)**	**0.296 (0.21-0.38)**
**Female:Symptoms**	**0.333 (0.22-0.46)**				
**Female:Vital**	0.507 (0.50-0.52)				
**Female:Vital + Symp**	**0.289 (0.19-0.40)**				
**Female:Thermal**		0.527 (0.51-0.56)	0.507 (0.50-0.53)	0.508 (0.50-0.53)	0.500 (0.50-0.50)
**Female:Thermal + Vital**		0.527 (0.51-0.56)	0.507 (0.50-0.52)	0.516 (0.50-0.54)	0.500 (0.50-0.50)
**Female:Thermal + Vital + Symp**		0.452 (0.33-0.57)	**0.344 (0.23-0.47)**	**0.303 (0.20-0.43)**	**0.306 (0.20-0.41)**

Table 2 Area under the ROC for all subjec analysis

AUC	No Imaging	Thermal Chest	Thermal Face	Thermal Back	Thermal Left-Right
**All:Symptoms**	**0.707 (0.62-0.79)**				
**All:Vital**	0.451 (0.36-0.54)				
**All:Vital + Symp**	**0.735 (0.66-0.81)**				
**All:Thermal**		0.581 (0.49-0.67)	0.505 (0.41-0.60)	0.357 (0.28-0.43)	0.307 (0.23-0.38)
**All:Thermal + Vital**		0.564 (0.47-0.66)	0.523 (0.43-0.61)	0.352 (0.27-0.43)	0.331 (0.25-0.41)
**All:Thermal + Vital + Symp**		**0.703 (0.62-0.78)**	**0.686 (0.60-0.77)**	***0.717 (0.64-0.80)***	**0.700 (0.62-0.78)**
**Male:Symptoms**	0.711 (0.60-0.82)				
**Male:Vital**	0.490 (0.38-0.60)				
**Male:Vital + Symp**	0.744 (0.65-0.84)				
**Male:Thermal**		0.609 (0.50-0.72)	0.519 (0.41-0.63)	0.354 (0.26-0.45)	0.309 (0.22-0.40)
**Male:Thermal + Vital**		0.608 (0.50-0.72)	0.546 (0.43-0.66)	0.338 (0.24-0.44)	0.336 (0.24-0.43)
**Male:Thermal + Vital + Symp**		0.743 (0.64-0.84)	0.713 (0.61-0.82)	*0.715 (0.61-0.82)*	0.717 (0.61-0.82)
**Female:Symptoms**	**0.707 (0.58-0.84)**				
**Female:Vital**	0.370 (0.22-0.52)				
**Female:Vital + Symp**	**0.719 (0.60-0.84)**				
**Female:Thermal**		0.532 (0.39-0.68)	0.475 (0.32-0.63)	0.357 (0.22-0.49)	0.307 (0.18-0.44)
**Female:Thermal + Vital**		0.480 (0.33-0.63)	0.475 (0.33-0.63)	0.374 (0.24-0.51)	0.328 (0.19-0.46)
**Female:Thermal + Vital + Symp**		0.621 (0.49-0.75)	**0.642 (0.51-0.78)**	***0.707 (0.59-0.83)***	**0.665 (0.53-0.80)**

Table 3 Sensitivity for selecting COVID-19 patients using all subjects.

Sensitivity		Thermal			
	No Imaging	Chest	Face	Back	Left-Right
**All:Symptoms**	0.593 (0.46-0.72)				
**All:Vital**	0.017 (0.00-0.09)				
**All:Vital + Symp**	0.729 (0.60-0.84)				
**All:Thermal**		0.102 (0.04-0.21)	0.017 (0.00-0.09)	0.017 (0.00-0.09)	0.000 (0.00-0.06)
**All:Thermal + Vital**		0.102 (0.04-0.21)	0.017 (0.00-0.09)	0.017 (0.00-0.09)	0.000 (0.00-0.06)
**All:Thermal + Vital + Symp**		0.525 (0.39-0.66)	0.672 (0.54-0.79)	*0.644 (0.51-0.76)*	0.678 (0.54-0.79)
**Male:Symptoms**	0.564 (0.40-0.72)				
**Male:Vital**	0.026 (0.00-0.13)				
**Male:Vital + Symp**	0.692 (0.52-0.83)				
**Male:Thermal**		0.154 (0.06-0.31)	0.026 (0.00-0.14)	0.026 (0.00-0.13)	0.000 (0.00-0.09)
**Male:Thermal + Vital**		0.154 (0.06-0.31)	0.026 (0.00-0.14)	0.026 (0.00-0.13)	0.000 (0.00-0.09)
**Male:Thermal + Vital + Symp**		0.564 (0.40-0.72)	0.658 (0.49-0.80)	*0.615 (0.45-0.77)*	0.641 (0.47-0.79)
**Female:Symptoms**	0.650 (0.41-0.85)				
**Female:Vital**	0.000 (0.00-0.17)				
**Female:Vital + Symp**	0.800 (0.56-0.94)				
**Female:Thermal**		0.000 (0.00-0.17)	0.000 (0.00-0.17)	0.000 (0.00-0.17)	0.000 (0.00-0.17)
**Female:Thermal + Vital**		0.000 (0.00-0.17)	0.000 (0.00-0.17)	0.000 (0.00-0.17)	0.000 (0.00-0.17)
**Female:Thermal + Vital + Symp**		0.450 (0.23-0.68)	0.700 (0.46-0.88)	*0.700 (0.46-0.88)*	0.750 (0.51-0.91)

Table 4 Balanced Error Rate for symptomatic subjects

BER		Thermal			
	No Imaging	Chest	Face	Back	Left-Right
**All:Symptoms**	**0.390 (0.32-0.46)**				
**All:Vital + Symp**	**0.419 (0.35-0.49)**				
**All:Thermal**		**0.406 (0.34-0.48)**	**0.442 (0.38-0.50)**	0.531 (0.49-0.58)	0.476 (0.42-0.54)
**All:Thermal + Vital + Symp**		**0.429 (0.35-0.50)**	0.506 (0.44-0.57)	0.455 (0.38-0.53)	0.479 (0.41-0.55)
**Male:Symptoms**	**0.343 (0.26-0.43)**				
**Male:Vital + Symp**	**0.363 (0.27-0.45)**				
**Male:Thermal**		**0.331 (0.24-0.42)**	0.440 (0.36-0.51)	0.502 (0.44-0.57)	0.438 (0.37-0.51)
**Male:Thermal + Vital + Symp**		**0.371 (0.28-0.46)**	0.503 (0.43-0.57)	0.442 (0.35-0.53)	0.479 (0.39-0.57)
**Female:Symptoms**	0.460 (0.33-0.57)				
**Female:Vital + Symp**	0.508 (0.39-0.62)				
**Female:Thermal**		0.555 (0.51-0.61)	0.456 (0.34-0.55)	0.583 (0.53-0.65)	0.541 (0.44-0.63)
**Female:Thermal + Vital + Symp**		0.550 (0.44-0.65)	0.502 (0.39-0.61)	0.478 (0.35-0.59)	0.489 (0.37-0.60)

Table 5 Area under de ROC curve for only symptomatic subjects

AUC		Thermal			
	No Imaging	Chest	Face	Back	Left-Right
**All:Symptoms**	0.526 (0.43-0.63)				
**All:Vital + Symp**	0.507 (0.41-0.61)				
**All:Thermal**		**0.593 (0.50-0.69)**	**0.595 (0.50-0.69)**	0.386 (0.30-0.48)	0.567 (0.48-0.66)
**All:Thermal + Vital + Symp**		**0.590 (0.50-0.68)**	0.466 (0.37-0.56)	0.561 (0.47-0.66)	0.570 (0.48-0.66)
**Male:Symptoms**	0.565 (0.44-0.69)				
**Male:Vital + Symp**	0.554 (0.43-0.68)				
**Male:Thermal**		**0.643 (0.53-0.76)**	0.575 (0.46-0.69)	0.452 (0.34-0.57)	0.594 (0.48-0.71)
**Male:Thermal + Vital + Symp**		**0.642 (0.53-0.76)**	0.431 (0.31-0.55)	**0.613 (0.50-0.73)**	0.580 (0.46-0.70)
**Female:Symptoms**	0.467 (0.30-0.63)				
**Female:Vital + Symp**	0.431 (0.26-0.60)				
**Female:Thermal**		0.463 (0.31-0.62)	0.629 (0.47-0.79)	0.244 (0.12-0.37)	0.531 (0.38-0.68)
**Female:Thermal + Vital + Symp**		0.493 (0.34-0.65)	0.504 (0.34-0.67)	0.459 (0.29-0.63)	0.549 (0.40-0.70)

Table 6 Sensitivity of the model for symptomatic subjects

Sensitivity	No Imaging	Thermal Chest	Thermal Face	Thermal Back	Thermal Left-Right
**All:Symptoms**	0.407 (0.28-0.54)				
**All:Vital + Symp**	0.373 (0.25-0.51)				
**All:Thermal**		0.373 (0.25-0.51)	0.207 (0.11-0.33)	0.068 (0.02-0.17)	0.186 (0.10-0.31)
**All:Thermal + Vital + Symp**		0.390 (0.27-0.53)	0.155 (0.07-0.27)	0.288 (0.18-0.42)	0.271 (0.16-0.40)
**Male:Symptoms**	0.436 (0.28-0.60)				
**Male:Vital + Symp**	0.436 (0.28-0.60)				
**Male:Thermal**		0.564 (0.40-0.72)	0.211 (0.10-0.37)	0.103 (0.03-0.24)	0.205 (0.09-0.37)
**Male:Thermal + Vital + Symp**		0.513 (0.35-0.68)	0.132 (0.04-0.28)	0.308 (0.17-0.48)	0.282 (0.15-0.45)
**Female:Symptoms**	0.350 (0.15-0.59)				
**Female:Vital + Symp**	0.250 (0.09-0.49)				
**Female:Thermal**		0.000 (0.00-0.17)	0.200 (0.06-0.44)	0.000 (0.00-0.17)	0.150 (0.03-0.38)
**Female:Thermal + Vital + Symp**		0.150 (0.03-0.38)	0.200 (0.06-0.44)	0.250 (0.09-0.49)	0.250 (0.09-0.49)
